# Analysis of early surgical indications and related factors in neonatal necrotizing enterocolitis

**DOI:** 10.3389/fped.2025.1571921

**Published:** 2025-05-20

**Authors:** Xianhui Shang, Huinan Wang, Yuanmei Liu, Xuefeng Yang, Lu Huang, Hao Fu, Luping Xiang, Shiyu Xu

**Affiliations:** ^1^ Department of Pediatric Surgery, Affiliated Hospital of Zunyi Medical University, Zunyi, China; ^2^ Department of Pediatric Surgery, Guizhou Children’s Hospital, Zunyi, Guizhou, China; ^3^ Department of General Surgery–Hernia and Pediatric Surgery, Northern Jiangsu People’s Hospital, Yangzhou University, Yangzhou, Jiangsu, China; ^4^ Department of Gastrointestinal Surgery, The Second Affiliated Hospital of Zunyi Medical University, Zunyi, Guizhou, China

**Keywords:** neonatal necrotizing enterocolitis, early surgery, peritonitis, IL-6, PCT, portal venous gas

## Abstract

**Objective:**

To explore early surgical indications and clinical predictive factors for neonatal necrotizing enterocolitis (NEC) to improve the prognosis of affected infants.

**Methods:**

A retrospective analysis was conducted on the clinical data of 146 infants diagnosed with NEC at the Affiliated Hospital of Zunyi Medical University from January 2015 to December 2020. The infants were divided into two groups: the surgical treatment group (56 cases) and the non-surgical treatment group (90 cases). Maternal perinatal conditions, general infant characteristics, clinical manifestations, comorbidities, laboratory tests, and imaging findings were statistically analyzed. Significant factors were further analyzed using multivariate logistic regression, and predictive indicators were assessed by the receiver operating characteristic (ROC) curve and Youden's index.

**Results:**

Statistically significant differences were observed between the two groups in birth weight, gestational age, abdominal wall erythema, absent bowel sounds, lethargy, fever, peritonitis, septic shock, metabolic acidosis, neonatal acute respiratory distress syndrome, and asphyxia (*P* < 0.05). No significant differences were found in maternal perinatal conditions, sex, feeding method, age at onset, abdominal distention, bloody stool, vomiting, gastric retention, apnea, neonatal pneumonia, neonatal hyperbilirubinemia, sepsis, electrolyte disturbances, or respiratory failure (*P* > 0.05). Laboratory and imaging markers such as prealbumin, IL-6, PCT, CRP, WBC, pneumoperitoneum, bowel wall gas, and portal venous gas showed statistically significant differences (*P* < 0.05). Multivariate logistic regression identified peritonitis (OR = 95.635), IL-6 (OR = 1.001), and portal venous gas (OR = 22.551) as independent risk factors for early surgery in NEC (*P* < 0.05). ROC curve analysis revealed that IL-6 (AUC = 0.875) and PCT (AUC = 0.798) demonstrated good predictive performance for early surgical intervention. The optimal cutoff values were 476 pg/ml for IL-6 (sensitivity 80.4%, specificity 85.6%) and 1.53 ng/ml for PCT (sensitivity 83.9%, specificity 70%).

**Conclusion:**

Peritonitis and portal venous gas are independent risk factors for early surgery in NEC. IL-6 and PCT are reliable predictive markers for determining the need for early surgical intervention in NEC.

## Introduction

Neonatal necrotizing enterocolitis (NEC) is one of the most common and life-threatening gastrointestinal emergencies in neonates, characterized by rapid progression and high mortality, with fatality rates reported as high as 35% (1). In recent years, advances in neonatal intensive care have significantly improved the survival rates of extremely low birth weight (ELBW) and ultra-low birth weight (ULBW) infants. However, this improvement has also been accompanied by a rising incidence of NEC, particularly among high-risk neonates (2).

The early clinical manifestations of NEC are typically nonspecific and insidious, making timely diagnosis and intervention extremely challenging. Many infants are diagnosed only after disease progression has occurred, and approximately 50% eventually require surgical treatment (3). However, the clinical course of NEC is highly variable, and significant heterogeneity exists in its pathophysiology among affected infants. Determining the optimal timing for surgical intervention remains a major clinical dilemma: delayed surgery can lead to extensive intestinal necrosis and worsened outcomes, whereas premature surgery may expose neonates to unnecessary risks.

Previous studies have attempted to identify predictive markers of surgical necessity in NEC, including abnormal vital signs, inflammatory biomarkers, and radiographic findings. Nevertheless, there is currently no widely accepted or quantitatively validated set of early predictors to guide surgical decision-making, particularly in the early stages of the disease when symptoms are subtle or atypical.

We hypothesize that a distinct set of clinical signs, laboratory parameters, and radiographic features observed at the time of NEC diagnosis may serve as independent risk factors to predict the need for surgical intervention.

To test this hypothesis, we conducted a retrospective analysis of neonates diagnosed with NEC and hospitalized at the Affiliated Hospital of Zunyi Medical University between January 2015 and December 2020. By comparing the clinical data of patients in the surgical and non-surgical groups, we aimed to identify potential early surgical indicators and predictive biomarkers that may assist in determining the optimal timing for surgical intervention, ultimately improving patient outcomes.

## Methods

### Data sources

A total of 219 neonates diagnosed with NEC were admitted to the Neonatology and Pediatric Surgery Departments of the Affiliated Hospital of Zunyi Medical University between January 2015 and December 2020. Of these, 146 neonates met the inclusion criteria. This study adhered to the principles of the 2013 Declaration of Helsinki. Ethics approval number: KLL-2023-570.

### Inclusion criteria

1. Met the diagnostic criteria for NEC (4).
2. Complete clinical data, including detailed records of maternal perinatal abnormalities, neonatal perinatal conditions, clinical manifestations, comorbidities, laboratory test results, abdominal x-ray findings, detailed surgical records, and discharge outcomes.

### Exclusion criteria

1. Neonates who died within 48 h of hospitalization and did not undergo adequate clinical assessment or imaging/Laboratory evaluation to determine surgical eligibility.
2. Neonates requiring surgery but whose families refused surgical treatment.
3. Neonates discharged against medical advice before meeting the discharge criteria for conservative treatment.
4. Incomplete medical records, including missing imaging or laboratory tests on the day of NEC diagnosis.

### Study design

A total of 146 neonates meeting the inclusion criteria were divided into a surgical treatment group (56 cases) and a non-surgical treatment group (90 cases). The surgical treatment criteria included a confirmed NEC diagnosis, failure of conservative treatment within 24–48 h, and progressive worsening of symptoms. In the surgical group, the extent of bowel lesions, surgical procedures, and outcomes were recorded. The following indicators were analyzed:

1. **Maternal perinatal conditions:** Including premature rupture of membranes, abnormal amniotic fluid, hypertensive disorders of pregnancy, placenta previa, amniotic cavity infection, and intrauterine distress.
2. **Neonatal general characteristics:** Including sex, birth weight, gestational age, feeding method, and age at onset.
3. **Clinical manifestations:** Including abdominal distention, vomiting, bloody stools, gastric retention, fever, lethargy, abdominal wall erythema, absent bowel sounds, and apnea.
4. **Comorbidities:** Including peritonitis, septic shock, metabolic acidosis, pneumonia, neonatal hyperbilirubinemia, sepsis, electrolyte disturbance, respiratory failure, neonatal respiratory distress syndrome (NRDS), and asphyxia.
5. **Laboratory tests:** Including prealbumin (PA), interleukin-6 (IL-6), procalcitonin (PCT), C-reactive protein (CRP), white blood cell count (WBC), and platelet count (PLT).
6. **Imaging findings:** Abdominal x-ray and ultrasound findings, including intestinal gas dilatation, bowel wall gas, bowel obstruction, interintestinal fluid, portal venous gas, and pneumoperitoneum.

### Analytical methods

Univariate analysis was performed on the indicators listed above. Variables with statistical significance were subjected to multivariate logistic regression analysis. Laboratory indicators were evaluated using receiver operating characteristic (ROC) curves and Youden's index. The area under the curve (AUC) was calculated to assess predictive performance. An AUC >0.7 indicated good predictive performance, while an AUC <0.5 indicated no predictive value. The laboratory indicator with the largest AUC was considered to have the best predictive performance. The optimal cutoff values for laboratory indicators were determined using Youden's index (calculated as sensitivity + specificity−1).

### Statistical analysis

Statistical analyses were conducted using SPSS software version 19.0. Categorical data were expressed as frequencies and percentages, while continuous data were presented as mean ± standard deviation (x¯ ± SD) for normally distributed variables or median (interquartile range) [M (P25–P75)] for non-normally distributed variables. Comparisons of categorical data between groups were performed using the χ^2^ test (or the χ^2^ correction formula for 1 ≤ T < 5). Normally distributed continuous variables were compared using the *t*-test, while non-normally distributed variables were analyzed using the Mann–Whitney *U*-test. Multivariate analysis was conducted using logistic regression, with results expressed as odds ratios (OR) and 95% confidence intervals (95% CI). A *P*-value <0.05 was considered statistically significant.

## Results

### General characteristics

Among the 219 neonates diagnosed with NEC, 146 cases were ultimately included in the analysis. A total of 73 cases were excluded for the following reasons:

1. Two neonates died within 48 h of admission due to rapid clinical deterioration and were excluded because they did not undergo sufficient clinical assessment, imaging, or laboratory evaluation to determine surgical eligibility.
2. Nineteen neonates who met surgical criteria were discharged against medical advice after their families declined surgical intervention, often opting for palliative care following a detailed explanation of the prognosis.
3. Twenty-eight neonates undergoing conservative treatment were discharged prematurely without meeting formal discharge criteria, typically due to family choice or socioeconomic constraints.
4. Twenty-four cases were excluded due to incomplete clinical data, including missing imaging or laboratory results.

### Intraoperative findings and outcomes in the surgical group

Of the 56 neonates in the surgical group, intestinal perforation was observed in 31 cases (55.36%). Among these, 15 cases involved small bowel perforation (13 ileal perforations), and 16 cases involved colonic perforation (11 transverse colonic perforations). Thirteen neonates presented with perforations in more than two intestinal sites. All 56 neonates exhibited varying degrees of intestinal necrosis (100%), with the following distributions:

1. Small bowel necrosis: 39 cases (30 ileal necroses, 5 jejunal necroses, and 6 cases of extensive small bowel necrosis).
2. Colonic necrosis: 32 cases (25 cases of ascending and transverse colon necrosis, and 18 cases of extensive colonic necrosis or necrosis in more than two sites).

### Surgical procedures

1. Resection of necrotic bowel with primary anastomosis: 8 cases.
2. Resection of necrotic bowel with stoma creation: 41 cases.
3. Intestinal decompression: 1 case.
4. Extensive total small bowel necrosis: 6 cases (5 families opted for withdrawal of treatment, and 1 case underwent palliative peritoneal drainage).

The shortest resected necrotic bowel length was 3 cm, while the longest was 70 cm. Fourteen neonates had necrosis exceeding 20 cm, and 6 neonates had necrosis exceeding 40 cm.

### Outcomes

Ten neonates died during hospitalization. Among them, six deaths were attributed to extensive intestinal necrosis, and four were due to complications. The remaining neonates were successfully treated and discharged.

### Comparison of clinical data between groups

Comparison of Maternal Perinatal Factors see Table 1

**Table 1:** Comparison of maternal perinatal factors between the surgical and non-surgical groups [*n* (%)].

Maternal perinatal factors	Surgical group (*n* = 56)	Non-surgical group (*n* = 90)	χ^2^ value	*P*-value
Premature rupture of membranes	11 (19.64)	16 (17.77)	0.080	0.778
Hypertensive disorders of pregnancy	2 (3.57)	9 (10.00)	1.229	0.268
Placenta previa	3 (5.35)	7 (7.77)	0.051	0.821
Abnormal amniotic fluid	5 (8.92)	8 (8.88)	0.000	1.000
Amniotic cavity infection	1 (1.78)	4 (4.44)	0.153	0.696
Intrauterine distress	2 (3.57)	2 (2.22)	0.000	1.000

### Comparison of general perinatal characteristics between the two groups

Gestational age and birth weight showed statistically significant differences between the surgical and non-surgical groups (*P* < 0.05) see Table 2.

**Table 2:** Comparison of general perinatal characteristics between the surgical and non-surgical groups [*n* (%)].

General characteristics	Study factors	Surgical group (*n* = 56)	Non-Surgical Group (*n* = 90)	χ^2^ value	*P*-value
Gender	Male	35 (62.50)	53 (58.88)	0.188	0.665
	Female	21 (37.50)	37 (41.11)		
Gestational age	<32 weeks	15 (26.78)	42 (46.66)	6.532	0.038*
	32 weeks∼	21 (37.50)	29 (32.22)		
	≥37 weeks	20 (35.71)	19 (21.11)		
Birth weight	<1,500 g	8 (14.29)	31 (34.44)	8.903	0.012*
	1,500 g∼	25 (44.64)	38 (42.22)		
	≥2,500 g	23 (41.07)	21 (23.33)		
Feeding method	Breastfeeding	12 (21.42)	14 (15.55)	0.813	0.367
	Non-breastfeeding	44 (78.57)	76 (84.44)		
Age at onset	<7 days	24 (42.85)	27 (30.00)	4.701	0.095
	7 days∼	19 (33.92)	27 (30.00)		
	≥14 days	13 (23.21)	36 (40.00)		

Note: *P* < 0.05 indicates a statistically significant difference between the surgical and non-surgical groups. *denotes variables with statistically significant differences.

### Comparison of clinical manifestations between the two groups

Significant differences were observed between the surgical and non-surgical groups in poor response, fever, abdominal wall erythema, and absent bowel sounds (*P* < 0.05) see Table 3.

**Table 3:** Comparison of clinical manifestations between the surgical and non-surgical groups [*n* (%)].

Clinical manifestation	Surgical group (*n* = 56)	Non-surgical group (*n* = 90)	χ^2^ value	*P*-value
Abdominal distention	55 (98.21)	83 (92.22)	1.376	0.241
Blood in stool	43 (76.78)	68 (75.55)	0.029	0.866
Vomiting	20 (35.71)	31 (34.44)	0.024	0.876
Gastric retention	16 (28.57)	25 (27.77)	0.011	0.917
Abdominal erythema	16 (28.57)	5 (5.55)	14.849	0.000*
Absent bowel sounds	54 (96.42)	74 (82.22)	6.446	0.011*
Poor response	52 (92.85)	64 (71.11)	9.999	0.002*
Fever	25 (44.64)	19 (21.11)	9.079	0.003*
Apnea	21 (37.50)	39 (43.33)	0.485	0.486

Note: *P* < 0.05 indicates a statistically significant difference between the surgical and non-surgical groups. *denotes variables with statistically significant differences.

### Comparison of comorbidities

Significant differences were observed between the surgical and non-surgical groups in peritonitis, septic shock, metabolic acidosis, and neonatal respiratory distress syndrome (*P* < 0.05) see Table 4.

**Table 4:** Comparison of complications between the surgical and non-surgical groups [*n* (%)].

Complications and comorbidities	Surgical group (*n* = 56)	Non-surgical group (*n* = 90)	χ^2^ value	*P*-value
Pneumonia	31 (55.35)	55 (61.11)	0.472	0.492
Jaundice	36 (64.28)	62 (68.88)	0.331	0.565
Peritonitis	46 (82.14)	4 (4.44)	92.548	0.000*
Septic shock	19 (33.92)	3 (3.33)	25.249	0.000*
Sepsis	16 (28.57)	18 (20.00)	1.42	0.233
Metabolic acidosis	24 (42.85)	14 (15.55)	13.364	0.000*
Neonatal respiratory distress syndrome	11 (19.64)	32 (35.55)	4.207	0.04*

Note: *P* < 0.05 indicates a statistically significant difference between the surgical and non-surgical groups. *denotes variables with statistically significant differences.

### Comparison of laboratory tests between the two groups

Significant differences were observed between the surgical and non-surgical groups for PA, IL-6, PCT, CRP, and WBC (*P* < 0.05) see Table 5.

**Table 5:** Comparison of laboratory indicators between the surgical and non-surgical groups.

Laboratory indicators	Surgical group (*n* = 56)	Non-surgical group (*n* = 90)	*t*/*Z*-value	*P*-value
PA (mg/L)	74.00 ± 33.04	85.41 ± 31.03	*t* = 2.108	0.037*
PLT (×10^9^/L)	224.54 ± 109.91	353.14 ± 124.95	*t* = 1.407	0.161
WBC (×10^9^/L)	6.16 (48–9.89)	9.57 (6.68–12.58)	*Z* = 3.505	0.000*
IL-6 (pg/ml)	1,223.50 (505.10–4,695.78)	86.35 (14.78–277.40)	*Z* = 7.618	0.000*
PCT (ng/ml)	6.05 (2.16–27.62)	0.76 (0.24–2.17)	*Z* = 6.037	0.000*
CRP (mg/L)	33.06 (5.71–54.64)	7.67 (0.77–32.15)	*Z* = 3.109	0.002*

Note: *P* < 0.05 indicates a statistically significant difference between the surgical and non-surgical groups. *denotes variables with statistically significant differences.

### Comparison of imaging findings

Significant differences were observed between the surgical and non-surgical groups in bowel wall gas, portal venous gas, and pneumoperitoneum (*P* < 0.05) see Table 6.

**Table 6:** Comparison of imaging findings between the surgical and non-surgical groups [*n* (%)].

x-ray findings	Surgical group (*n* = 56)	Non-surgical group (*n* = 90)	χ^2^ value	*P*-value
Bowel dilatation with air	46 (82.14)	72 (80.00)	0.102	0.749
Intestinal OBSTRUCTION	7 (12.50)	3 (3.33)	3.223	0.073
Bowel wall pneumatosis	14 (25.00)	2 (2.22)	18.355	0.000*
Portal venous gas	11 (19.64)	1 (1.11)	13.355	0.000*
Intramural gas	1 (1.78)	2 (2.22)	0.000	1.000
Pneumoperitoneum	12 (21.42)	0 (0.00)	18.268	0.000*

Note: *P* < 0.05 indicates a statistically significant difference between the surgical and non-surgical groups. *denotes variables with statistically significant differences.

### Multivariate logistic regression analysis

Peritonitis (OR = 95.635, 95% CI: 25.312\u2013361.339), IL-6 (OR = 1.001, 95% CI: 1.000\u20131.001), and portal venous gas (OR = 22.551, 95% CI: 1.802\u2013282.190) were identified as independent factors for predicting early surgical indications in NEC (*P* < 0.05) see Table 7.

**Table 7:** Logistic regression analysis of factors related to NEC surgery.

Variable	β value	SE value	Wald value	*P*-value	OR value	OR value 95% CI	
						Lower limit	Upper limit
Peritonitis	4.561	0.678	45.216	0.000	95.635	25.312	361.339
IL-6	0.001	0.000	7.542	0.006	1.001	1.000	1.001
Portal venous gas	3.116	1.289	5.841	0.016	22.551	1.802	282.190
Constant	−2.949	0.479	37.917	0.000	0.052	–	–

### ROC curve analysis of laboratory indicators

**Figure 1:**
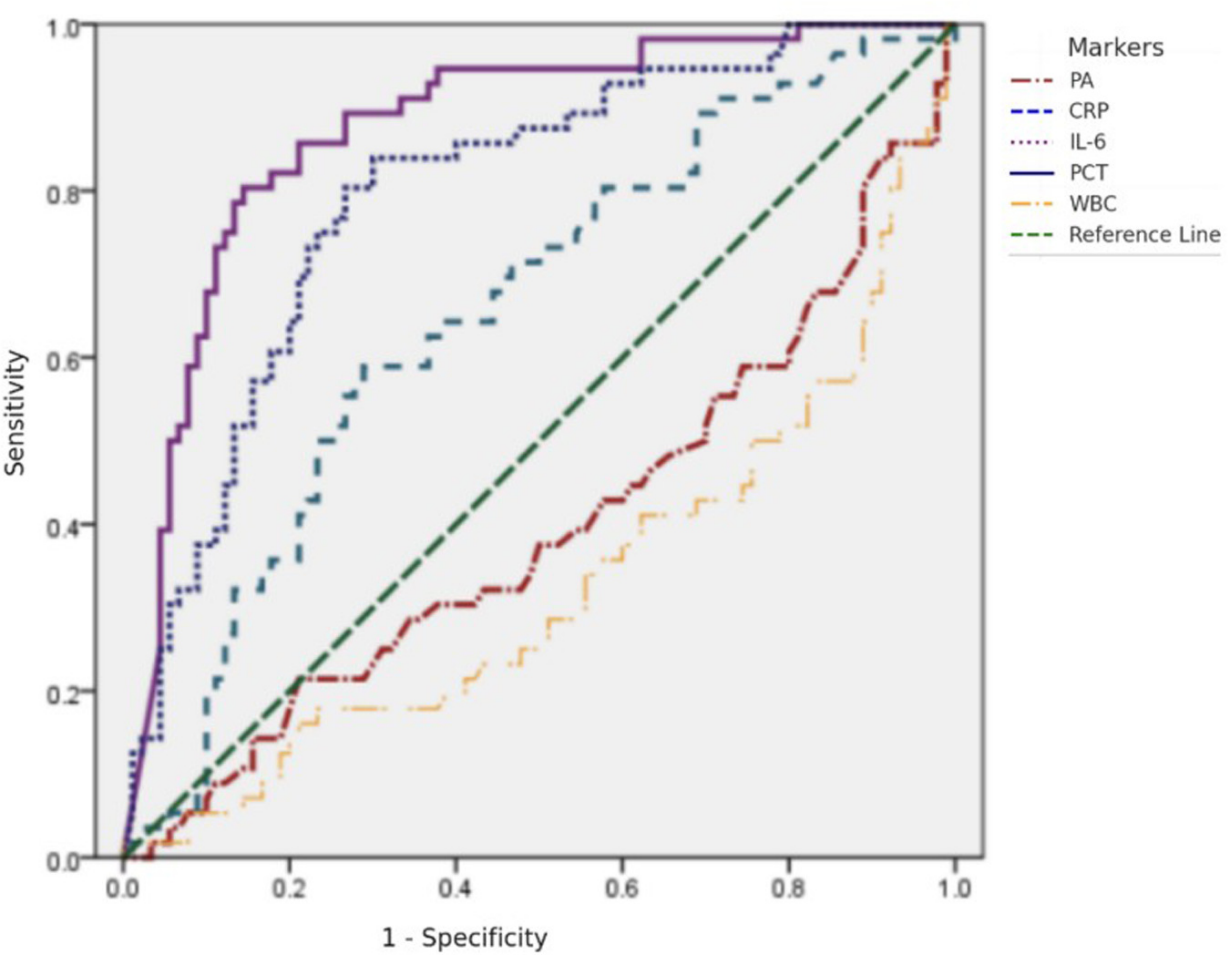
ROC Curve for the Predictive Efficacy of WBC, PA, PCT, CRP, and IL-6 for NEC Surgery.

The results of the ROC curve analysis and Youden index indicated that IL-6 (AUC = 0.875) and PCT (AUC = 0.798) demonstrated good predictive performance for early surgical intervention in NEC, with IL-6 showing the best predictive efficacy, followed by PCT. CRP (AUC = 0.653) had moderate predictive efficacy, while WBC and PA had no predictive value. The optimal cutoff value for predicting surgical timing was 476 pg/ml for IL-6, 1.53 ng/ml for PCT, and 27.515 mg/L for CRP. Among these indicators, IL-6 had the highest specificity, PCT had the highest sensitivity, and CRP showed poor sensitivity and specificity see Figure 1 and Table 8.

**Table 8:** Comparison of diagnostic efficacy of CRP, IL-6, and PCT for predicting NEC surgery.

Diagnostic efficacy	CRP (mg/L)	IL-6 (pg/ml)	PCT (ng/ml)
Optimal cut-off value	27.515	476	1.53
Sensitivity	58.9%	80.4%	83.9%
Specificity	71.1%	85.6%	70%
Youden index	0.300	0.659	0.539
AUC	0.653	0.875	0.798

## Discussion

Currently, most scholars agree that the optimal timing for NEC surgery is when there is intestinal ischemia and necrosis but before overt perforation occurs (5). However, accurately determining this window—especially in the absence of definitive signs such as pneumoperitoneum or clinical deterioration—remains a key challenge in neonatal surgical decision-making. Although peritonitis and portal venous gas are recognized indicators for surgical intervention, they may appear only in advanced stages of NEC, limiting their utility for early decision-making. Therefore, this study aimed to identify earlier, quantifiable clinical and biochemical markers that can supplement traditional indicators and better inform surgical timing.

In our cohort, peritonitis, portal venous gas, and elevated levels of IL-6 and PCT were independently associated with the need for surgical intervention. While peritonitis and portal venous gas are not novel indicators, their independent statistical significance in this analysis reaffirms their importance in clinical practice. Rather than proposing new criteria, our intention was to assess whether newer biomarkers—particularly IL-6—can serve as early warning indicators in patients with subtle or equivocal signs, thus supporting more timely intervention.

The predictive role of IL-6 has been previously reported (6), and our study reinforces its utility in this context. Although the odds ratio (OR) for IL-6 was 1.001, this reflects its use as a continuous variable across a wide range of concentrations (from <100 to >5,000 pg/ml). As such, a per-unit OR may underestimate its actual clinical relevance when applied to larger magnitude changes. Statistical consultation confirmed the robustness of this result, and IL-6 showed the highest predictive performance (AUC = 0.875) among all parameters tested.

PCT also emerged as a useful biomarker, with an optimal cutoff of 1.53 ng/ml and good sensitivity and specificity (83.9% and 70%, respectively), consistent with earlier reports (7). IL-6 and PCT may jointly enhance early risk stratification in NEC. CRP was also elevated in surgical cases but showed relatively modest predictive value, aligning with existing literature (8, 9).

Previous studies have reported that NEC infants with intestinal perforation are predominantly preterm and of low birth weight, likely due to immature intestinal development and impaired perfusion, placing them at higher risk for requiring surgical intervention (2, 10). Consistent with prior studies (11–14), lower gestational age and birth weight were significantly more frequent in the surgical group, reaffirming their role as risk factors for disease progression and surgical intervention. Conversely, although 82.2% of NEC infants were not breastfed, feeding mode did not differ significantly between groups, suggesting a stronger association with disease onset rather than progression to surgical NEC (15, 16).

With respect to imaging, bowel wall gas and portal venous gas—but not all forms of bowel dilation—were significantly associated with surgery, in line with prior consensus (9, 17). Notably, 20% (3/15) of patients with surgically confirmed perforation had no radiographic evidence of pneumoperitoneum, emphasizing the limitation of imaging alone and supporting a multimodal approach incorporating biochemical and clinical findings.

## Limitations

This study has several limitations. First, its single-center, retrospective design and moderate sample size limit generalizability and introduce potential selection bias. Second, the retrospective nature inherently limits the ability to establish causality, and the inclusion of known surgical indicators such as peritonitis may introduce confirmation bias. Third, the inflammatory markers evaluated—CRP, PCT, and IL-6—are non-specific and may be elevated in other infections or systemic inflammatory states. Therefore, interpretation must occur in conjunction with clinical and radiological assessments. Lastly, while IL-6 showed statistical significance, its low OR value should be interpreted cautiously due to the nature of the continuous variable; further prospective validation is needed.

## Conclusion

This study identified peritonitis, portal venous gas, elevated IL-6, and elevated PCT as independent risk factors for early surgical intervention in neonates with NEC. Among these, IL-6 demonstrated the highest predictive accuracy, suggesting its value as an objective biomarker for surgical timing. Additionally, low gestational age and birth weight remain essential considerations for early risk identification.

These findings underscore the importance of a comprehensive, multimodal evaluation that incorporates clinical signs, laboratory markers, and imaging to guide timely and individualized surgical decision-making. While these results support preliminary clinical utility, prospective multicenter studies involving dynamic monitoring and disease-specific biomarkers are warranted to refine early surgical criteria and improve outcomes in NEC.

## Data Availability

The original contributions presented in the study are included in the article/Supplementary Material, further inquiries can be directed to the corresponding author.
